# hsa-miR-875-5p inhibits tumorigenesis and suppresses TGF-β signalling by targeting USF2 in gastric cancer

**DOI:** 10.1186/s12967-022-03253-6

**Published:** 2022-03-07

**Authors:** Shenshuo Gao, Zhikai Zhang, Xubin Wang, Yan Ma, Chensheng Li, Hongjun Liu, Changqing Jing, Leping Li, Xiaobo Guo

**Affiliations:** 1 Department of Gastroenterological Surgery, Shandong Provincial Hospital, Cheeloo College of Medicine, Shandong University, Jinan, Shandong 250021 China; 2 Department of Gastroenterological Surgery, Shandong Provincial Hospital Affiliated to Shandong First Medical University, Jinan, Shandong 250021 China

**Keywords:** miR-875-5p, USF2, Gastric cancer, TGF-β

## Abstract

**Background:**

Gastric cancer (GC) is one of the most common malignancies, and an increasing number of studies have shown that its pathogenesis is regulated by various miRNAs. In this study, we investigated the role of miR-875-5p in GC.

**Methods:**

The expression of miR-875-5p was detected in human GC specimens and cell lines by miRNA qRT–PCR. The effect of miR-875-5p on GC proliferation was determined by Cell Counting Kit-8 (CCK-8) proliferation and 5-ethynyl-2′-deoxyuridine (EdU) assays. Migration and invasion were examined by transwell migration and invasion assays as well as wound healing assays. The interaction between miR-875-5p and its target gene upstream stimulatory factor 2(USF2) was verified by dual luciferase reporter assays. The effects of miR-875-5p in vivo were studied in xenograft nude mouse models. Related proteins were detected by western blot.

**Results:**

The results showed that miR-875-5p inhibited the proliferation, migration and invasion of GC cells in vitro and inhibited tumorigenesis in vivo. USF2 was proved to be a direct target of miR-875-5p. Knockdown of USF2 partially counteracted the effects of miR-875-5p inhibitor. Overexpression of miR-875-5p could inhibit proliferation, migration and invasion and suppress the TGF-β signalling pathway by downregulating USF2.

**Conclusions:**

MiR-875-5p can inhibit the progression of GC by directly targeting USF2. And in the future, miR-875-5p is expected to be a potential target for GC diagnosis and treatment.

## Background

Gastric cancer (GC) is one of the most common malignant tumours of the digestive tract due to excessive proliferation of gastric epithelial cells. Worldwide, GC ranks fourth in morbidity and second in mortality [1]. Due to the lack of effective biomarkers for early diagnosis, GC patients often develop to an advanced stage when they are diagnosed, and their 5-year survival rate is less than 30% [2]. Early diagnosis of GC can avoid this deterioration and improve survival in patients with GC. To improve the prognosis of GC patients, more effective biomarkers for early diagnosis are needed. Therefore, there is an urgent need to understand the basic mechanism of GC tumorigenesis and development and to find effective biological targets. Although changes in oncogenes and tumour suppressor genes have been reported in GC, the pathogenesis of GC and the complex molecular mechanisms and signal transduction pathways involved in the progression and development of GC have not been fully studied, which limits effective biological targets and clinical treatment [3]. Therefore, the exact molecular mechanisms of GC pathogenesis still need to be fully clarified.

MicroRNAs (miRNAs) are a class of small, highly conserved, noncoding single-stranded RNA molecules encoded by endogenous genes 18–22 nucleotides in length that directly target the 3′ untranslated region (3′-UTR) of genes by binding to certain sequence-specific sites [4]. The silencing complex induced by miRNA prevents the translation or promotes direct degradation of mRNA, resulting in a decrease in the expression of these genes [5]. There are various regulatory relationships between miRNAs and mRNAs, affecting epigenetics, RNA stability and translation [6]. MiRNAs play an important role in cell proliferation, metastasis, differentiation, apoptosis and development [7]. It has been shown that miRNAs are abnormally expressed in cancer tissues compared to normal tissues [8, 9]. There are still many unknown details about the role of miRNAs in human cancer, and further research is needed.

MiR-875-5p is dysregulated in many diseases, including gestational diabetes mellitus [10], liver fibrosis [11], lung cancer [12], esophageal cancer [13], hepatocellular carcinoma [14], prostate cancer [15], colorectal carcinoma [16] and thyroid cancer [17]. These results suggest that miR-875-5p plays a role in the occurrence and development of tumours. In a previous study, we found that the expression of miR-875-5p was decreased in GC [18], but its role in GC remains unclear. This study aims to explore the effect of miR-875-5p on GC and its potential mechanism.

Upstream stimulatory factor (USF) was first identified in HeLa nuclear extract which binds to elements in the major late promoter of adenovirus (E-box) and stimulates gene transcription [19, 20]. Two USF peptides with apparent molecular weights of 43 and 44 kDa were obtained from HeLa cells, called USF1 and USF2, respectively [21, 22]. USF2 is a basic helix-loop-helix (BHLH) transcription factor encoded by heterodimeric and overlapping genes [23]. Isotypes produced by splicing show different transcriptional activities in certain promoter environments [24, 25]. Although it is widely believed that USF2 expression and relative abundance vary [26], USF2 interacts synergistically with other factors in tissue and stimulus-specific transcriptional regulation [27, 28]. Studies have shown that USF2 plays a promoting role in the development of breast cancer, colorectal cancer and lung cancer [29–32].

This study aims to explore the biological function of miR-875-5p in GC and its potential mechanism. We demonstrate for the first time that miR-875-5p directly targets the 3′-UTR of human USF2 mRNA. Here, we report that miR-875-5p is indeed inhibited in primary GC compared to matched adjacent normal gastric tissues and that the 3′-UTR of human USF2 mRNA is actually the target of miR-875-5p. Further experiments also showed that miR-875-5p inhibited the expression of USF2 at the mRNA level by directly binding to the 3′ untranslated region of USF2, and thus affected the TGF-β signalling pathway, thus inhibiting the proliferation, migration and invasion of GC cells.

## Materials and methods

### Tissue samples

Human GC tissues and paired adjacent normal tissues were collected from 30 patients with GC who underwent radical gastrectomy at the Department of General Surgery, Shandong Provincial Hospital, Cheeloo College of Medicine, Shandong University, China. After surgical excision, the sample was quickly frozen in liquid nitrogen for experiments. This study was approved by the ethics committee of Shandong Provincial Hospital. The collection of gastric cancer and para-cancer tissue and its use was approved by the Institutional Review Board of the Shandong Provincial Hospital, Cheeloo College of Medicine, Shandong University, Shandong, China.

### Cell culture

Six gastric cancer cell lines (AGS, BGC-823, HGC-27, MGC-803, SGC-7901, MKN-45) and GES-1 cell lines were purchased from Institute of Biochemistry and Cell Biology, Chinese Academy of Sciences (Shanghai, China). Cells were cultured in RPMI 1640 supplemented with 10% fetal bovine serum (10% FBS), 100 U/mL penicillin, and 100 mg/mL streptomycin (Gibco, Grand Island, NY, USA). And cells were incubated in moist air at 37 °C and 5% CO2.

### Cell transfection

Hsa-miR-875-5p mimics and mimics negative control, hsa-miR-875-5p inhibitor and inhibitor negative control were purchased from GenePharma (Shanghai, China). At least 24 h before transfection, the cells were cultured in complete medium without antibiotics. Cells were planted in six-well plates, when the cell fusion degree reached 50–70%, the cells were washed with 1 × PBS (PH7.4), 50 nM miR-875-5p mimics or miR-mimic NC and 100 nM miR-875-5p inhibitor or miR-inhibitor NC were transfected into AGS and MKN-45 cells through Lipofectamine™2000 (Invitrogen). Si-USF2 and si-NC were purchased from Genomeditech (Shanghai, China) and transfected as above.

### RNA extraction and qRT-PCR

Total RNA was extracted from GC tissues and cells using Trizol reagent (Takara, Japan) according to the manufacturer’s instructions. The cDNA was synthesized using the Evo M-MLV RT Premix for qPCR reagent (AG, China). QRT-PCR was performed on Roche LightCycler 480 II fluorescent quantitative PCR instrument with qPCR SYBR Green Pro Taq HS Master Mix(AG). β-actin was used as the endogenous control. The primers used in this study are as follows: USF2 forward: 5′-AAAGGAGGGATCCTGTCCAA-3′, USF2 reverse: 5′-CAGGGCGTTCTCATTCTTCA-3′; β-actin forward: 5′-GCATCGTCACTGGGGAC-3′ and β-actin reverse: 5′-ACCTGG CCGTCAGGCAGCTC-3′. In addition, unchained curves were used to evaluate specific amplification. QRT-PCR reaction procedures are as follows: 95 ℃ for 30 s, 40 cycles at 95 °C 5 s and 60 °C 30 s. All procedures were carried out in triplicate and relative expression was calculated by the 2^−ΔΔCT^ method.

### miRNA qRT-PCR

Total RNA was extracted as mentioned above. According to the manufacturer's instructions, using the Mir-X miRNA first-Strand Synthesis Kit (Takara, Japan), RNA (2 µg) was converted to cDNA. PCR reaction was performed using TB Green® Premix Ex TaqTM II (Takara, Japan), and U6 was used as the endogenous control. The primers of miR-875-5p and U6 were purchased from RiboBio (RiboBio Co., Ltd, Guangzhou, China), the primer sequences used in this study were as follows; has-miR-875-5p forward:5′-GCGGGCGGTATACCTCAGTTTTAT-3′, reverse 5′-ATCCAGTGCAGGGTCCGAGG-3′; U6 forward: 5′-CTCGCTTCGGCAGCACA-3′; U6 reverse: 5′-AACGCTTCACGAATTTGCGT-3′. 2^−ΔΔCt^ method was used to calculate the relative expression level. All procedures were also performed in triplicate.

### Protein extraction and Western blot

The protein was extracted 72 h after transfection. The cells were flushed with cold PBS, then RIPA buffer (Beyotime, Shanghai, China) containing PMSF (SolarBio, Beijing, China) and phosphatase inhibitors (SolarBio, Beijing, China) was used for cracking. The protein concentration was calculated using the BCA Protein Assay Kit (Beyotime, Shanghai, China) and the protein was separated by SDS-PAGE using a 10% and 12% polyacrylamide gel (20 μg per sample). Proteins are transferred to the immobilon-NC membrane by electrotransfer. The imblotted membrane was sealed in 5% skimmed milk diluted with TBST, and then incubated with appropriate primary antibodies (anti-USF2, anti-p21, anti-p57, anti-Cyclin D1, anti-ZEB1, anti-E-cadherin, anti-Vimentin, anti-TGF-β1, anti-smad2, anti-phospho-smad2, anti-smad3, anti-phospho-smad3 and anti-GADPH obtained from CST) at 4 °C for 12 h.

### Dual-luciferase reporting assay

Dual luciferase assay was used to further verify the targeting relationship of miR-875-5p and USF2. Bioinformatics analysis predicted that the possible binding site of miR-875-5p was the 3′UTR site 595–601 of USF2 mRNA.

AGS and MKN-45 cells were grown in 1640 medium containing 10% FBS and transfected with psicheck2-Husf2-3′ UTR reporter plasmid and human miR-875-5p mimics or inhibitor using Lipofectamine™2000. After 48 h of culture, luciferase activity was detected to determine whether the microRNA binds and regulates USF2.

### CCK8 assay

Cell growth was measured using cell proliferation reagent CCK-8 (MCE). Cells were inoculated into a 96-well plate (Corning Costar, Corning, NY) at a concentration of 2.0 × 10^3^ per well, as per manufacturer’s instructions, and 10 μL CCK8 was added to each well at harvest. One hour after CCK8 was added, cell viability was determined by measuring the absorbance of the transformed dye at 450 nM.

### EdU experiment

The transfected cells were seeded into a 96-well plate at a concentration of 1.0 × 10^4^/well. The EdU solution was diluted with cell complete medium in a ratio of 1000:1 to prepare an appropriate amount of 50 μM EdU medium. Add 100 μL 50 μm EdU medium to each well and incubate for 2 h. Then follow the manufacturer’s instructions for the operations. The results were observed using a high-content imaging system.

### Transwell migration/invasion assay

MKN-45 and AGS cells grew to approximately 70% confluence in RPMI 1640 containing 10% fetal bovine serum and then transfected. After 24 h, the cells were digested by trypsin and then washed once with PBS. To measure cell migration, culture inserts with an 8 μm aperture (Transwell; CoStar, High Wycombe, UK) were placed in the holes of the 24-well plate and the upper and lower chambers were separated. In the lower chamber, add RPMI 1640 containsing 20% FBS, 600 μl. Then, serum-free medium containing 5 × 10^4^ cells was added to the upper compartment for migration assay, and 1 × 10^5^ cells were used for matrix gel invasion assay (Matrigel Basement Membrane Matrix; BD, NJ, USA). After incubation at 37 °C and 5% CO2 for 24 h, the cells in the upper compartment were removed with a cotton swab. The cells that invaded the base membrane of the inserts were fixed in 4% paraformaldehyde for 10 min, stained with 1% crystal violet for 20 min, rinsed in PBS, and examined microscopically. Each experiment was performed at least three times.

### Wound healing assay

To evaluate the motility of GC cells. A total of 1 × 10^6^ cells/wells were inoculated in a 6-well plate and cultured overnight, then transfected with miR-875-5p mimics or NC, miR-875-5p inhibitor or NC and si-USF2 or NC. 8 h later, the confluent cell monolayers were scraped with a sterile pipette head and the plates were washed twice with PBS and adding fresh serum-free medium immediately. An image of the plate is taken under a microscope. The clearance size was analyzed with Image J software.

### Tumorigenesis in nude mice

BALB/c nude mice (male, 4–6 weeks old, 16–20 g) were purchased from Beijing Vital River Laboratory Animal Technology Co., Ltd (Beijing, China). All animal experiments were conducted in accordance with the Guidelines for the Care and Use of Laboratory Animals of Provincial Hospital Affiliated to Shandong University. MiR-875-5p NC or miR-875-5p mimics were transfected into MKN-45 cells, and 5 × 10^5^ MKN-45 cells in logarithmic growth phase were suspended in 100 μL phosphate buffer, and then seeded subcutaneously into the right axillary of nude mice. The experiments were divided into two groups on average (miR-875-5p NC group and miR-875-5p mimics group, n = 6), tumor size was monitored by measuring length (L) and width (W) with a vernier caliper every 4 days, and volume was calculated using the following formula: (L × W^2^)/2. The mice were fed for 28 days. Tumors were sacrificed and collected. Tumor volume and weight was measured for analysis. Animal experiments conformed to the standards set by the Declaration of Helsinki, and were approved by the ethics review Committee of Shandong Provincial Hospital affiliated to Shandong University, Shandong, China.

### Immunohistochemical staining of xenograft tumors tissue

Tumor sections were incubated overnight with commercial rabbit polyclonal antibodies against USF2 at 1:50 dilution at 4 °C. Then, the slices were diluted with horseradish peroxidase (HRP) antibody (1:100; Invitrogen, Thermo Fisher, US), conjugated at room temperature for 2 h and then covered with DAB (SP kit (rabbit streptavidin–biotin method detection system), ZSGB-BIO, China). After rinsing the colored plates with water for a period of time, they were soaked in hematoxylin and dyed, then dehydrated and sealed. Subsequently, the results of IHC staining were scored by evaluating the extent and intensity of staining in 5 fields of view using a microscope (Tissue FAXS Systems, Austria) at × 200 magnification. The staining intensity was divided into four grades: no staining, score 0; pale yellow, score 1; pale brown, score 2; and dark brown, score 3. The positive expression area was also classified into five categories: < 5%, score 0; 6–25%, score 1; 26–50%, score 2; 51–75%, score 3; and 76–100%, score 4. The multiplication of intensity and area scores was used as the final USF2 expression score. All slides were scored by two independent pathologists from Shandong Provincial Hospital who had no knowledge of the grouping and treatment of the slides. When there were discrepancies between the two pathologists, the mean score was used.

### Statistical analysis

The results are presented as means ± SD. Statistical significance was measured by multiple comparisons using Student’s t-test with a significance level of *p* < 0.05.

## Results

### miR-875-5p is downregulated in human GC tissues and cell lines

**Fig. 1:**
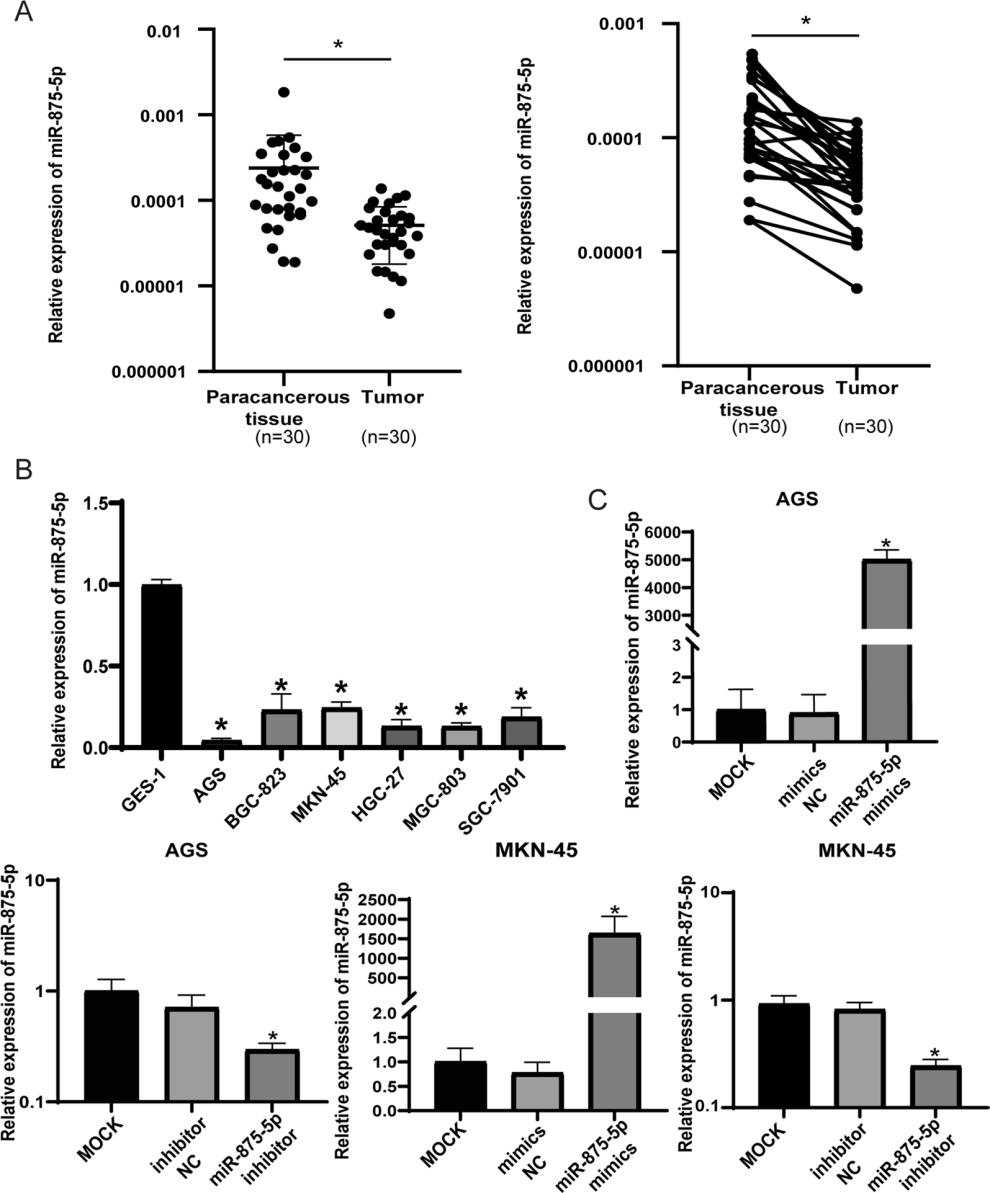
Expression of miR-875-5p in GC tissues and GC cells. **A** The miRNA qRT-PCR was used to examine the expression level of miR-875-5p in 30 pairs of human GC tissues and adjacent normal tissues. **B** The miRNA qRT-PCR was used to examine the expression level of miR-875-5p in GES-1 and GC cells. **C** The efficacy of transfection was verified in cells transfected with miR-875-5p mimics and miR-875-5p inhibitor. All experiments are repeated three times. *p < 0.05; Data expressed as mean ± SD

To confirm the abnormal expression of miR-875-5p in GC tissues, 30 pairs of GC tissues and matched adjacent normal gastric tissues were collected, and the relative expression of miR-875-5p was examined by miRNA qRT–PCR. As shown in Fig. 1A, the expression of miR-875-5p in human GC tissues was lower than that in the matched adjacent normal gastric tissues (*p* < 0.05). The expression of miR-875-5p was further examined by miRNA qRT–PCR in normal gastric mucosal epithelial cells (GES-1) and GC cell lines (AGS, BGC-823, MKN-45, HGC-27, MGC-803 and SGC-7901). As shown in Fig. 1B, the expression of miR-875-5p in GC cell lines was lower than that in GES-1 cells (*p* < 0.05). These data suggest that miR-875-5p is downregulated in GC tissues and GC cell lines.

To further investigate the biological role of miR-875-5p in GC cells, miR-875-5p mimics or inhibitor were transfected into AGS and MKN-45 cells. As shown in Fig. 1C, the expression level of miR-875-5p increased after transfection with miR-875-5p mimics and decreased after transfection with inhibitor in AGS and MKN-45 cells. However, compared with normal gastric tissue cells, the expression of miR-875-5p in GC cells was already decreased, so ectopic expression was more effective than further downregulation.

### miR-875-5p inhibits the proliferation of GC cell lines

**Fig. 2:**
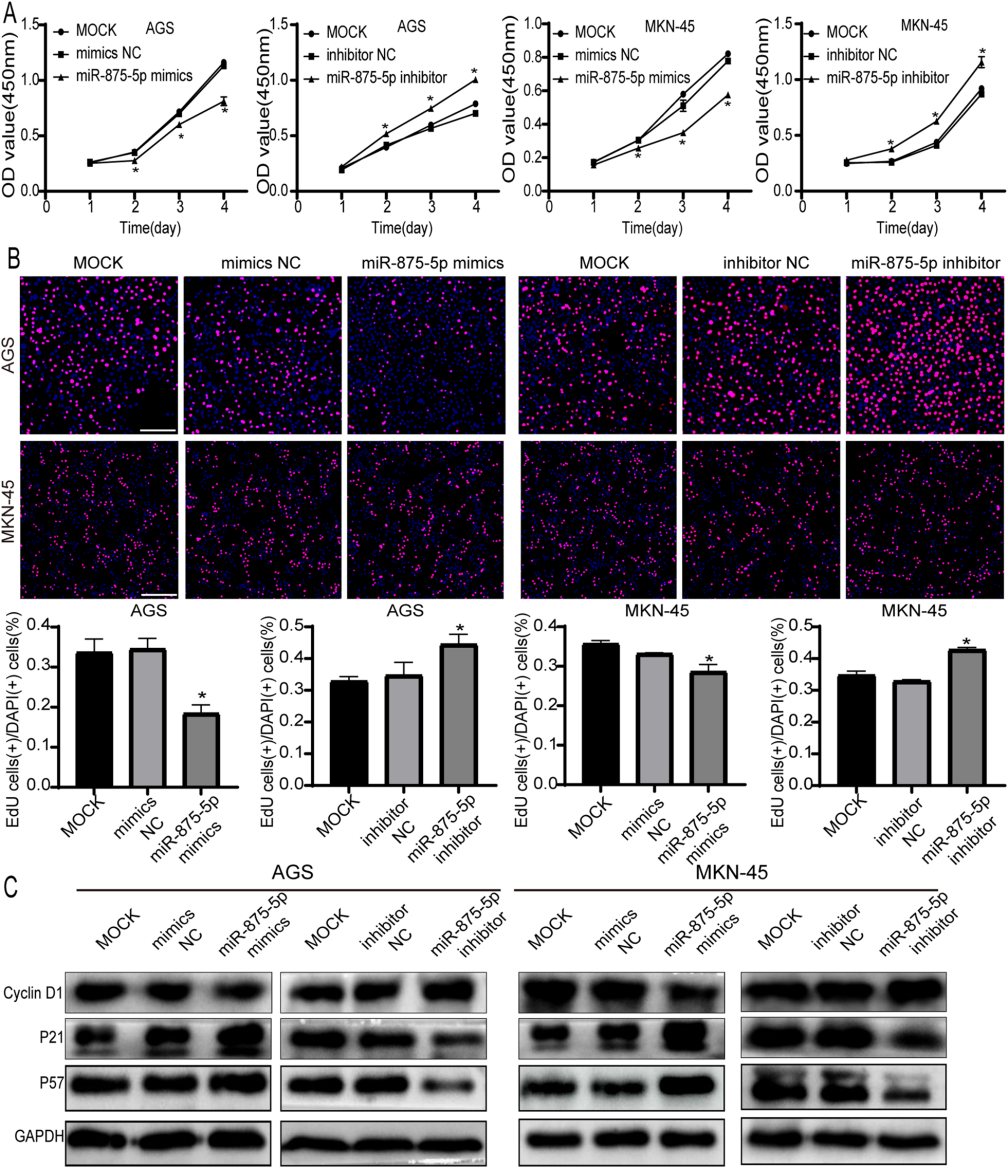
miR-875-5p inhibited the proliferation of GC cells in vitro. **A** CCK8 assays were used to evaluate the effect of miR-875-5p on the proliferation of AGS and MKN-45 cells. **B** EdU assays showed the effect of overexpression or silencing of miR-875-5p on the growth of GC cells. The white scale bar, 100 μm. **C** Western blotting showed the levels of proliferation-related proteins (Cyclin D1, P21 and P57) in AGS and MKN-45 cells with miR-875-5p overexpression or miR-875-5p silencing and their negative control cells. All experiments were repeated three times. *p < 0.05; Data expressed as mean ± SD

CCK-8 proliferation assay results showed that overexpression of miR-875-5p significantly inhibited the proliferation of GC cells, and knockdown of miR-875-5p significantly promoted the proliferation of GC cells (*p* < 0.05, Fig. 2A). The EdU assay results indicated that the overexpression of miR-875-5p significantly inhibited proliferation in AGS and MKN-45 cells, while the knockdown of miR-875-5p generated the opposite effects in AGS and MKN-45 cells (*p* < 0.05, Fig. 2B). These results demonstrate that miR-875-5p inhibits the proliferation of GC cells. Cyclin D1 is highly expressed and promotes tumorigenesis in numerous tumours [33, 34]. In our research, the protein expression of cyclin D1 was repressed by overexpression of miR-875-5p (Fig. 2C). Our study revealed that overexpression of miR-875-5p is a mechanism for the upregulation of p57 (a cyclin-dependent kinase inhibitor) and p21 (a cell cycle inhibitor) levels in GC cell lines (AGS and MKN-45) (Fig. 2C). These results suggest that miR-875-5p can inhibit proliferation in GC cells.

### miR-875-5p inhibits the migration and invasion of GC cell lines

**Fig. 3:**
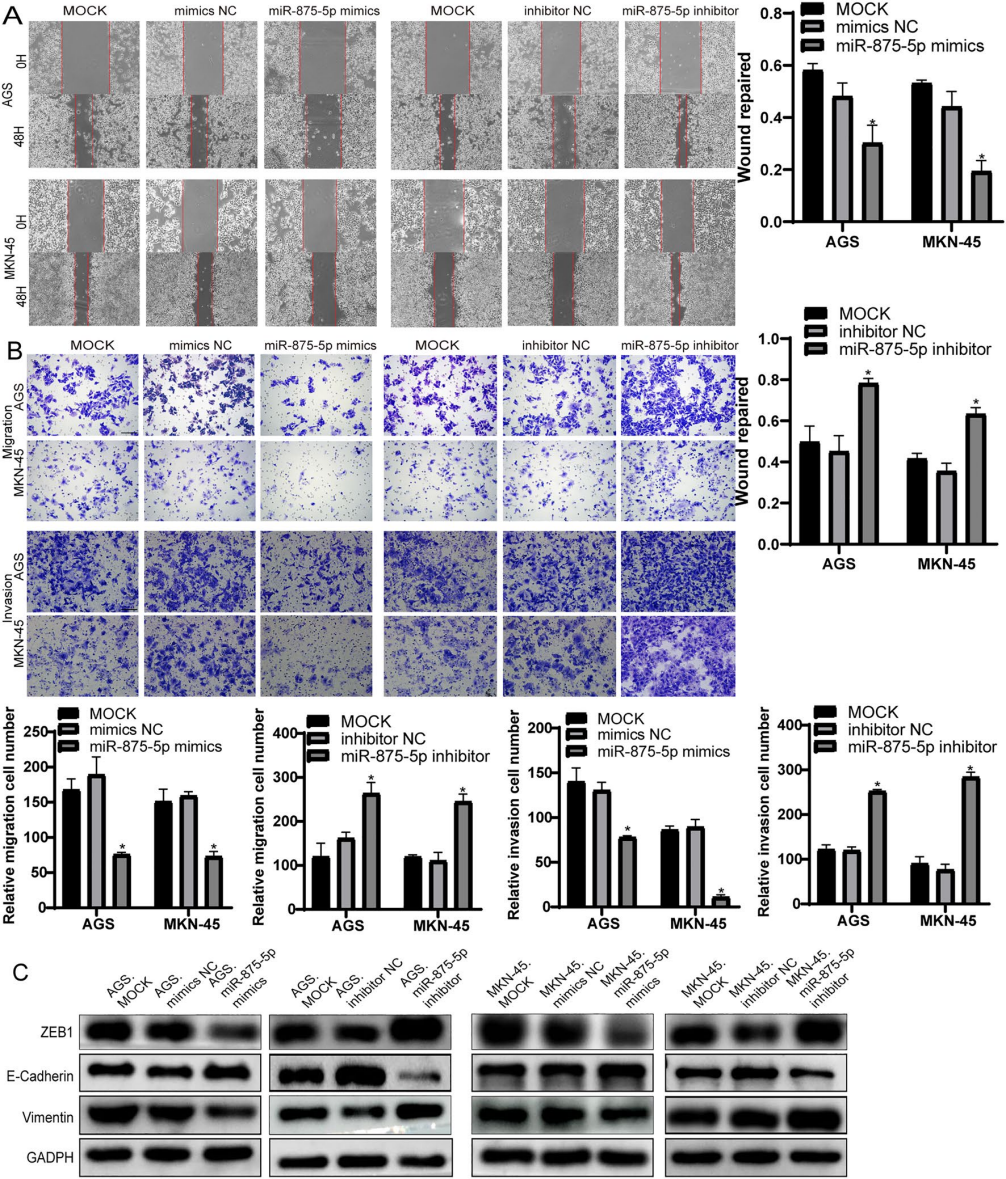
miR-875-5p inhibited migration and invasion in vitro and induced associated protein changes. **A** The wound healing assays were performed to study the changes of miR-875-5p overexpression and silencing on the migration ability of AGS and MKN-45 cells. The percentage of wound closure were calculated and compared. **B** The transwell assays were performed to detect the migration and invasion of AGS and MKN-45 cells. The black scale bar, 100 μm. **C** Western blot was performed to detect the expressions of EMT-related proteins in AGS and MKN-45 cells after transfection. All experiments were repeated three times. *p < 0.05; Data expressed as mean ± SD

The inhibitory effect of miR-875-5p in GC cells was further investigated. In the wound healing assay, overexpression of miR-875-5p reduced the migration rate of AGS and MKN-45 cells. In contrast, knockdown of miR-875-5p significantly accelerated AGS and MKN-45 cell migration (Fig. 3A). The effect of miR-875-5p on GC cells migration and invasion was examined by cell migration and matrix invasion assays. As shown in Fig. 3B, the number of penetrating AGS and MKN-45 cells in the miR-875-5p mimic group was also reduced compared with that in the control group, while silencing miR-875-5p showed the opposite effect in AGS and MKN-45 cells. In addition, the overexpression of miR-875-5p significantly inhibited the expression of epithelial-mesenchymal transition (EMT)-related proteins in GC cells, whereas the knockdown of miR-875-5p significantly promoted the expression of EMT-related proteins in GC cells (Fig. 3C). These experiments suggest that miR-875-5p inhibits the migration and invasion of GC cells.

### USF2 is the direct target of miR-875-5p

**Fig. 4:**
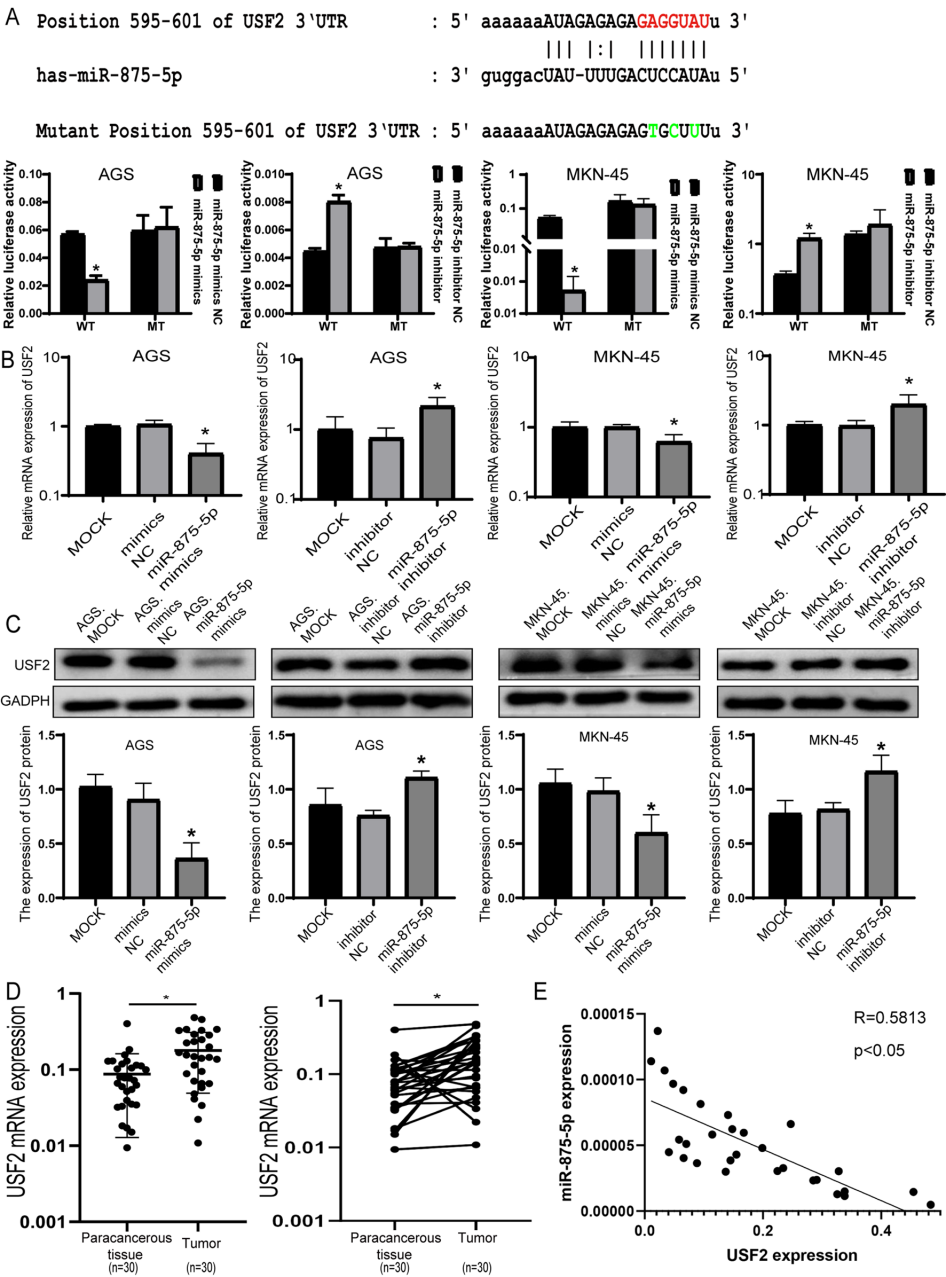
USF2 was confirmed to be a direct target of miR-875-5p. **A** The luciferase reporter assays were performed to confirm that miR-875-5p directly bound to the 3′-UTR of USF2. Putative miR-875-5p binding site in the human USF2 3′-UTR and luciferase constructs with the wild-type and mutant miR-875-5p target sequences. The red colours indicate the wild sequences of the USF2 3′-UTR. The green colours indicate the mutant sequences of the USF2 3′-UTR. The luciferase activity results were analysed in cells treated with miR-875-5p mimics or negative control and miR-875-5p inhibitor or negative control. **B** The expression level of USF2 mRNA was detected by qRT–PCR in AGS and MKN-45 cells after transfection. **C** The expression level of USF2 protein was detected by western blot in AGS and MKN-45 cells after transfection. **D** Expression of USF2 mRNA in GC tissues and matched normal gastric tissues. **E** USF2 showed a negative correlation with miR-875-5p in 30 GC tissue samples detected by qRT–PCR. All experiments were repeated three times. *P < 0.05; Data expressed as the mean ± SD

The target of miR-875-5p was predicted by TargetScan (http://www.targetscan.org/vert_71/), miRmap (https://mirmap.ezlab.org/) and starBase (https://starbase.sysu.edu.cn/). According to the predictions of three miRNA bioinformatics websites and our previous research [35], we found that USF2, an oncogene in many malignancies, may be one of the target genes of miR-875-5p. To confirm whether miR-875-5p regulates USF2, we performed double luciferase assays. Reported analysis showed that ectopic miR-875-5p expression significantly inhibited the luciferase activity of the wild-type (WT) USF2 3′UTR in AGS and MKN-45 cells but did not inhibit the luciferase activity of the mutated (MUT) USF2 3′UTR. In contrast, inhibition of miR-875-5p significantly increased luciferase activity of AGS and MKN-45 cells in the wild-type (WT) USF2 3′UTR, while there was little change in luciferase activity of mutated (MUT) USF2 3′UTR (Fig. 4A). Consistent with the reported analysis, we found that ectopic miR-875-5p reduced USF2 mRNA and protein levels. As well as, the mRNA and protein expression level of USF2 increased after knocking down miR-875-5p in AGS and MKN-45 cells (Fig. 4B, C). In addition, USF2 mRNA expression was detected in 30 GC tissue samples and matched gastric normal tissues, and the results showed that USF2 was highly expressed in GC tissues (Fig. 4D) and miR-875-5p was negatively correlated with USF2 (Fig. 4E).

Collectively, these data demonstrated that miR-875-5p directly bound to the USF2 3′-UTR and negatively regulated the expression of USF2.

### USF2 promotes proliferation, migration and invasion in GC cells

**Fig. 5:**
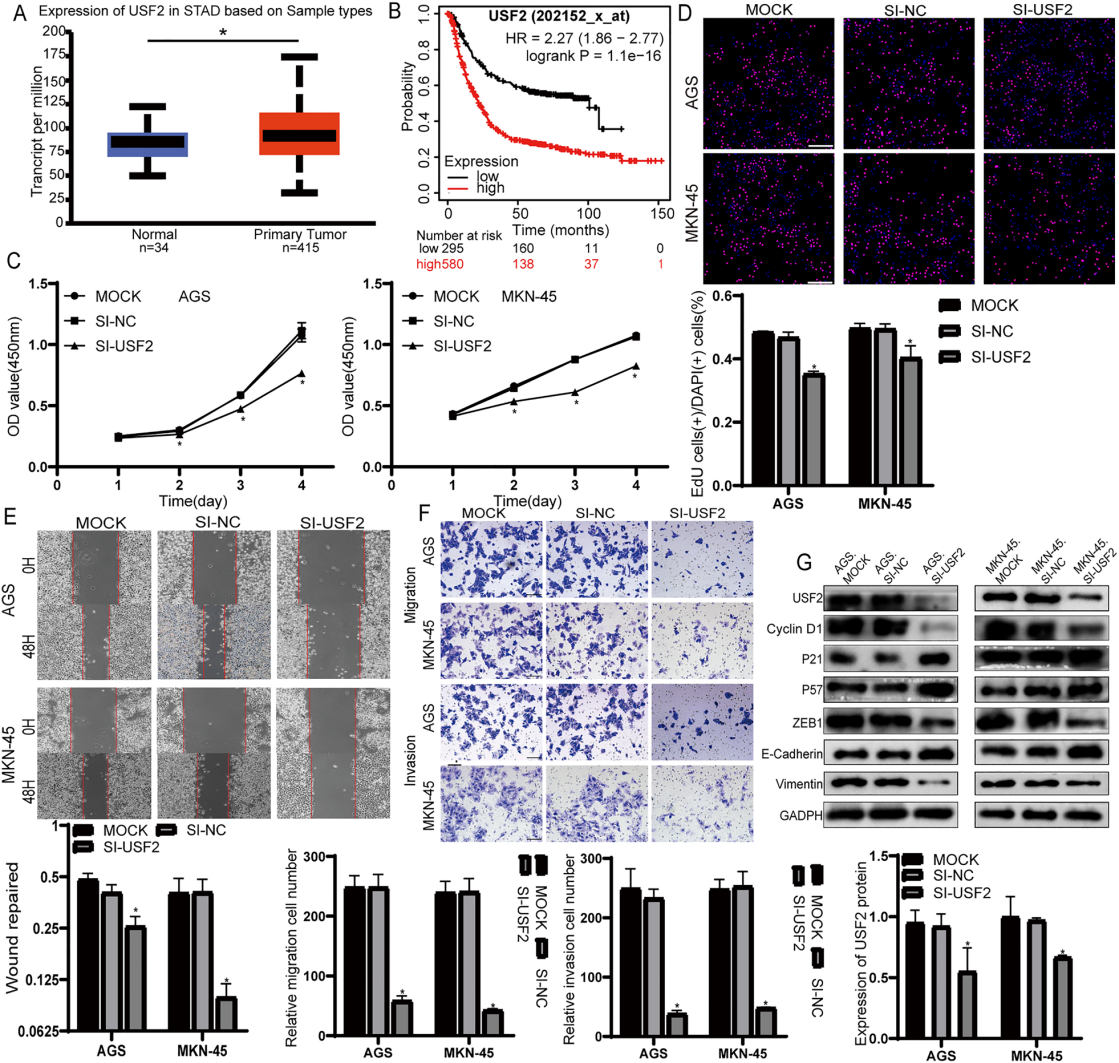
USF2 affected the proliferation, migration and invasion of GC cells. **A** Relative expression of USF2 in GC tissues and normal tissues from TCGA. **B** The survival curve of USF2 in GC patients based on K-M plotter database (http://www.kmplot.com, data from TCGA). (p < 0.001). **C** CCK8 assays were performed to detect the proliferation of AGS and MKN-45 cells transfected with si-USF2 or si-NC. **D** EdU assays were performed to detect the proliferation of AGS and MKN-45 cells transfected with si-USF2 or si-NC. **E** Wound-healing assays were used to detect migration ability of AGS and MKN-45 cells transfected with si-USF2 or si-NC. **F** Transwell migration and invasion assays were performed to determine the migration and invasion ability of AGS and MKN-45 cells transfected with si-USF2 or si-NC. **G** Western Blot detected the expression of associated proteins. All experiments were repeated three times. *p < 0.05; Data expressed as mean ± SD

According to the TCGA database, USF2 is upregulated and associated with poor prognosis in GC (Fig. 5A, B). To detect the potential effect of USF2 on the proliferation, migration and invasion of AGS and MKN-45 cells. AGS and MKN-45 cells were transfected with USF2 siRNA or negative control. CCK-8 and EdU assays showed that USF2 knockdown significantly inhibited the proliferation of AGS and MKN-45 cells (Fig. 5C, D). The wound healing assays showed that knockdown of USF2 significantly inhibited the migration of AGS and MKN-45 cells (Fig. 5E). In addition, Transwell assays showed that knockdown of USF2 significantly inhibited the migration and invasion of AGS and MKN-45 cells (Fig. 5F). We also demonstrated that knockdown of USF2 caused changes in the related proteins (Fig. 5G).

### MiR-875-5p inhibits proliferation, migration and invasion in GC cells by targeting USF2

**Fig. 6:**
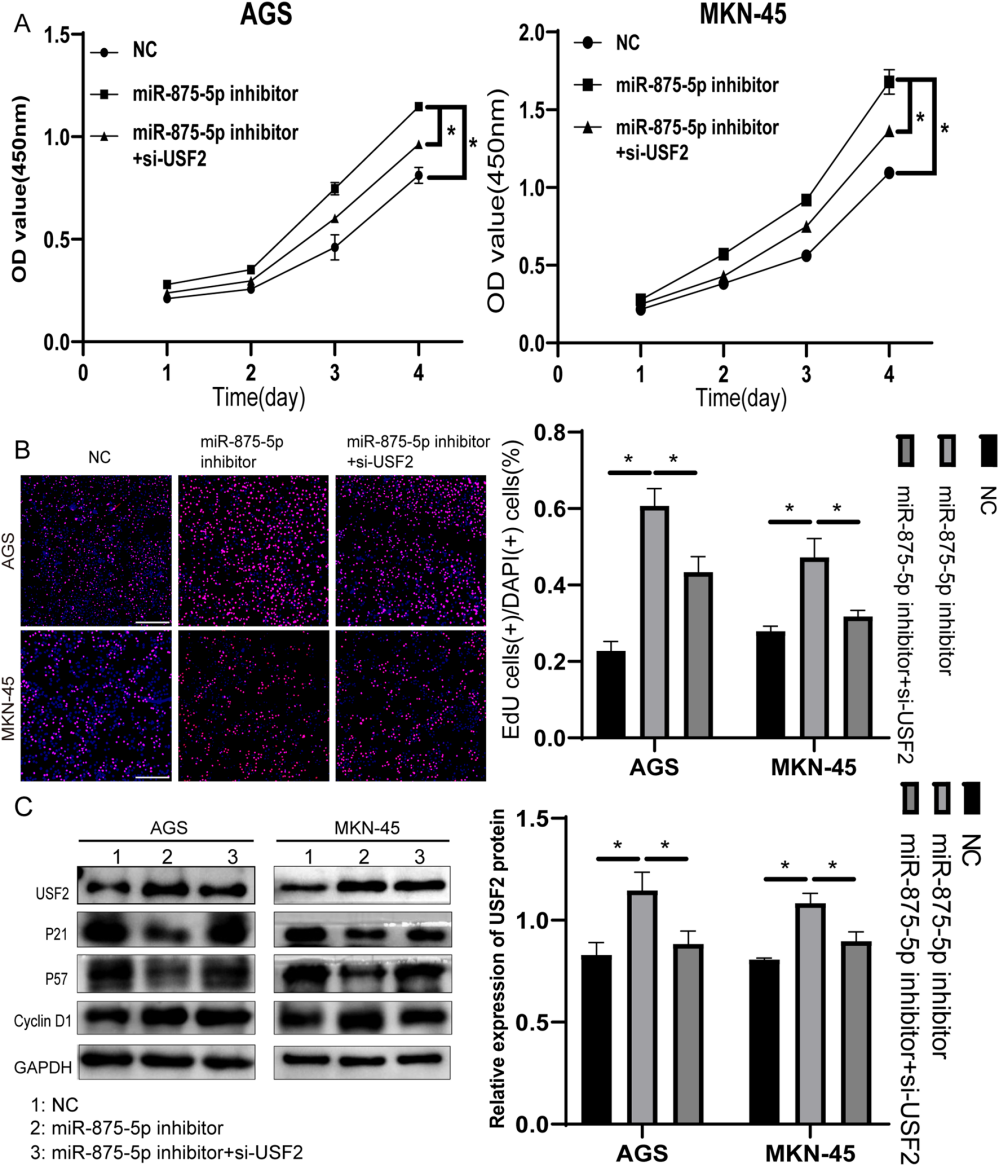
The effect of miR-875-5p silencing on GC cells was partially offset by USF2 knockdown. **A** CCK-8 assays were performed to determine that USF2 knockdown could partly counteract miR-875-5p silencing-induced proliferation in AGS and MKN-45 cells. **B** EdU assays were performed to determine that USF2 knockdown could partly counteract miR-875-5p silencing-induced proliferation in MKN-45 and AGS cells. **C** Western blot analysis showed changes in related proteins, such as USF2, Cyclin D1, P21 and P57, in AGS and MKN-45 cells after transfection. All experiments were repeated three times. *p < 0.05; Data expressed as mean ± SD

**Fig. 7:**
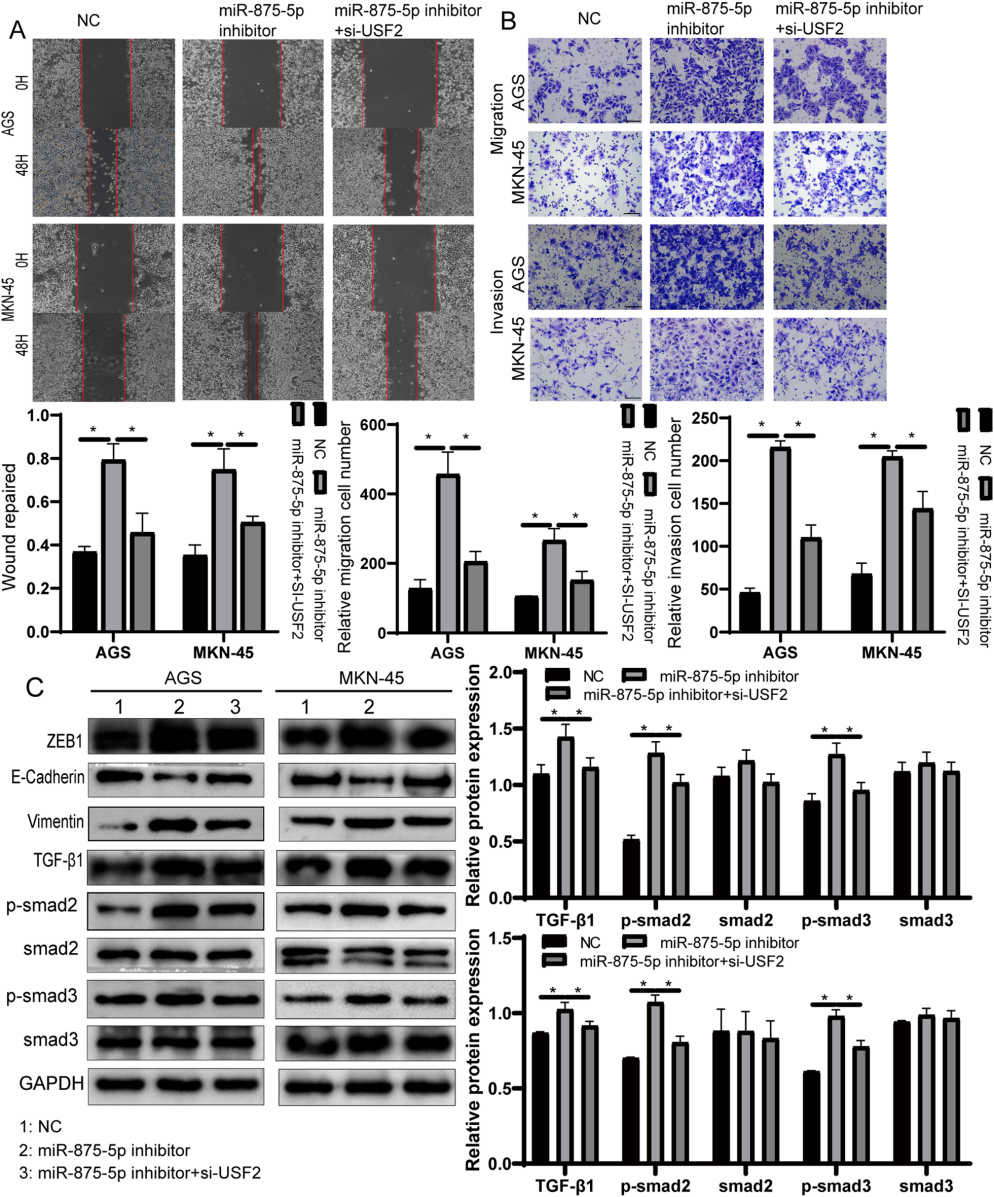
The effect of miR-875-5p silencing on GC cells was partially offset by USF2 knockdown. **A** Changes in cell migration were examined by wound healing assays in AGS cells and MKN-45 cells. **B** Transwell assays were used to examine the migration and invasion in AGS and MKN-45 cells. **C** Western blotting was performed to detect the expression of EMT-related proteins and TGF-β signalling pathway-related proteins in AGS and MKN-45 cells. All experiments were repeated three times. *p < 0.05; Data expressed as mean ± SD

We demonstrated that miR-875-5p overexpression inhibited proliferation, migration and invasion in GC cells and inhibited USF2 protein expression through mRNA degradation. The results were opposite when miR-875-5p was knocked down. Next, we will further confirm that miR-875-5p inhibited proliferation, migration and invasion in GC by regulating USF2. AGS and MKN-45 cells were cotransfected with miR-NC + si-NC, miR-875-5p inhibitor + si-NC, and miR-875-5p inhibitor + si-USF2. Through CCK-8 and EdU assays, we found that downregulation of USF2 in AGS and MKN-45 cells partially offset the effect of miR-875-5p silencing on proliferation (Fig. 6A, B). Proliferation-associated proteins were detected by western blot (Fig. 6C). Through wound healing assays and transwell migration and invasion assays, we found that knockdown of USF2 in AGS and MKN-45 cells partially offset the effect of miR-875-5p silencing on migration and invasion (Fig. 7A, B). EMT-related proteins were detected by western blotting (Fig. 7C). These findings suggest that miR-875-5p inhibits GC cell proliferation, migration and invasion by directly targeting USF2.

### MiR-875-5p suppresses the TGF-β signalling pathway by targeting USF2

To examine the mechanisms by which miR-875-5p and USF2 inhibit GC proliferation, migration and invasion, we investigated whether miR-875-5p mediated these effects by affecting the TGF-β signalling pathway. Western blotting was used to examine TGF-β1, Smad2, p-Smad2, Smad3 and p-Smad3. As shown in Fig. 7C, the silencing of miR-875-5p showed a significant increase in TGF-β1, p-Smad2 and p-Smad3 in GC cells compared to the control group. However, there was no significant difference in Smad2 and Smad3 expression. These effects can be partially counteracted by downregulating USF2 expression in miR-875-5p inhibitor cells. These results suggest that miR-875-5p suppresses the TGF-β signalling pathway by targeting USF2.

### MiR-875-5p suppresses the growth of GC in vivo

**Fig. 8:**
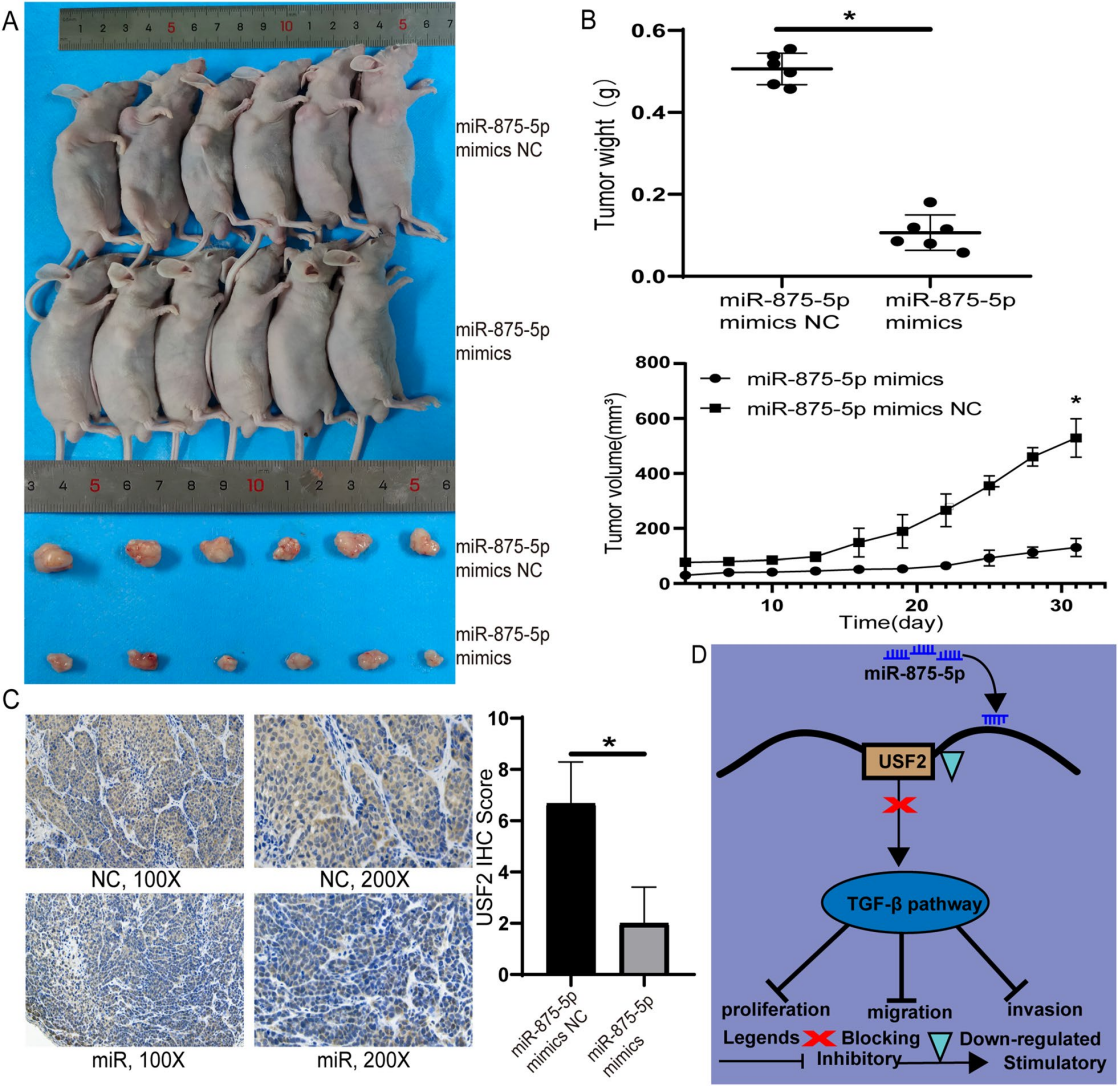
miR-875-5p inhibited tumour formation in vivo. **A** Xenograft tumours were obtained from different groups of nude mice injected with MKN-45 cells transfected with miR-875-5p NC and miR-875-5p mimics. **B** The tumour growth curve and tumour weight were significantly different between the miR-875-5p mimic group and the miR-875-5p NC group. **C** The expression of USF2 in xenograft tumours was determined by immunohistochemistry. **D** Proposed model of miR-875-5p regulating USF2 and affecting the TGF-β signalling pathway. *P < 0.05; Data expressed as mean ± SD

To demonstrate the effect of miR-875-5p on tumour growth in vivo, miR-875-5p mimics transfected cells were injected into the flanks of nude mice, and miR-NC transfected cells served as negative controls. As shown in Fig. 8, compared with the control group, the volume and weight of subcutaneous tumours in nude mice in the miR-875-5p-mimics group were significantly reduced (P < 0.05). In addition, immunohistochemistry was used to investigate the expression levels of USF2. Compared with the control group, USF2 protein levels were significantly reduced in the miR-875-5p-mimics group (Fig. 8C). miR-875-5p inhibited the growth of GC cells in vivo. Taken together, our results indicate that miR-875-5p inhibits GC progression and suppresses the TGF-β signalling pathway by targeting USF2 (Fig. 8D).

## Discussion

Abnormal expression of miRNAs plays an important role in the occurrence and development of tumours [36–38]. Previous studies have shown that miRNAs can bind to specific sites of certain sequences to directly target the 3′-UTR of genes and prevent their translation or directly promote degradation of mRNA, thus participating in the occurrence and development of tumours [9, 39, 40]. As a newly discovered tumour-related miRNA, the role of miR-875-5p in GC and the specific mechanism of its role remain unclear. In this study, we analysed the expression level of miR-875-5p in GC tissues and normal tissue adjacent to cancer, GC cell lines and GES-1 cells and predicted the target genes. In CCK-8 and EdU experiments, the overexpression of miR-875-5p inhibited the proliferation of GC cells and affected the expression of the proliferation-related proteins Cyclin D1, P21 and P57. The knockdown of miR-875-5p showed the opposite results, indicating that miR-875-5p played an inhibitory role in the proliferation of GC cells. Tumour spread and metastasis are affected by the migration and invasion ability of tumour cells, both of which are inhibited by miR-875-5p in vitro. Transwell migration and invasion experiments showed that the overexpression of miR-875-5p inhibited the migration and invasion of GC cells. In addition, the expression of EMT-related proteins ZEB-1, E-cadherin and Vimentin were affected, ZEB-1 and Vimentin were high and E-cadherin were low when miR-875-5p was knocked down. This result was reversed when miR-875-5p was overexpressed. Experiments in nude mice demonstrated that the overexpression of miR-875-5p inhibited the tumorigenicity of GC cells in vivo, and this series of experiments showed that miR-875-5p has an inhibitory effect on GC, suggesting that miR-875-5p may be a potential therapeutic target for GC patients. Western blotting and dual luciferase reporter analysis showed that USF2 is the target gene of miR-875-5p and is negatively correlated with miR-875-5p. Our study also showed that miR-875-5p directly binds to the 3′-UTR of USF2 to inhibit its expression and suppress the TGF-β signalling pathway.

To elucidate the mechanism of the effect of miR-875-5p on proliferation, migration and invasion. Bioinformatics analysis predicted the putative target of miR-875-5p in GC cells. According to our previous studies [32], USF2 was selected among the candidate target genes. USF2 plays an important role in various cellular processes, especially in the genesis and progression of tumours [41–43]. However, the role of USF2 in different tumours is contradictory, suggesting that it is either a tumour promoter or an inhibitor [29–31, 44]. The role of USF2 in GC has not been studied. Therefore, it is necessary to study its expression and role in GC. Compared with normal gastric tissue, the expression of USF2 in GC tissue was significantly increased. In the CCK-8 experiment and EdU experiment, the OD value and the proportion of proliferating cells in the si-USF2 group were significantly decreased, indicating that the proliferation of GC cells was inhibited after USF2 knockdown. The wound healing assays and transwell migration and invasion experiments showed that the migration and invasion ability of GC cells was inhibited after USF2 knockout. Dual luciferase experiments further confirmed that miR-875-5p could directly bind to the USF2 3'-UTR and degrade USF2 mRNA. In addition, knockdown of USF2 partially offset the promoting effect of miR-875-5p silencing on proliferation, migration and invasion in GC cells. In conclusion, our results indicate that the inhibitory effect of miR-875-5p in GC is mediated by downregulation of USF2.

Previous studies have shown that the TGF-β signalling pathway is associated with tumour proliferation and metastasis [45–48]. USF2 has been reported to downregulate Smurf1 and Smurf2, thereby regulating the TGF-β pathway in breast cancer [30]. Therefore, we studied the TGF-β pathway changes induced by miR-875-5p expression in GC. Western blot results showed that the expression of TGF-β1, phospho-Smad2 and phospho-Smad3 increased after knockdown of miR-875-5p, while the expression of Smad2 and Smad3 remained unchanged. On the basis of miR-875-5p knockdown, the expression of TGF-β1, phospho-Smad2 and phospho-Smad3 decreased slightly after knockdown of USF2. The expression of Smad2 and Smad3 remained unchanged. These results suggest that the decrease of miR-875-5p can activate the TGF-β signalling pathway by upregulating USF2. In other words, miR-875-5p can be used as a negative regulator of the TGF-β pathway to inhibit the proliferation, migration and invasion of GC. However, it cannot be ruled out that miR-875-5p can also influence other signalling pathways to exert anticancer effects. Some studies in mouse cells have shown that miR-875-5p targets gli1. Their experiments found that icariin treatment decreased GLI1 and SMO expression and increased PTCH1 expression in HSCs, suggesting that icariin inhibited hedgehog signalling pathway activation in hedgehog signalling pathway cells. Treatment with the miR-875-5p inhibitor partially offset these effects. These results indicate that miR-875-5p can activate the hedgehog signalling pathway. In addition, in human GC cells, studies have found that Notch3 is the target gene of miR-491-5p/miR-875-5p, and the two are negatively correlated, while Notch3 positively regulates PHLDB2, which in turn affects the Akt-mTOR pathway. Therefore, miR-875-5p may affect the occurrence and development of GC through a variety of pathways.

## Conclusion

Our study showed that miR-875-5p was significantly downregulated in GC tissues and GC cell lines. As a key inhibitor of the growth and metastasis of GC cells, miR-875-5p inhibited the progression of GC and suppressed the TGF-β signalling pathway by targeting USF2. In the future, miR-875-5p is expected to be used as a potential therapeutic target for GC therapy.

## Data Availability

The datasets used and/or analyzed during the current study are available from the corresponding author on reasonable request.

## Abbreviations

USF2: Upstream stimulatory factor2
miRNA: MicroRNA
siRNA: Small interfering RNA
3′-UTR: 3′- Untranslated region
qRT-PCR: Quantitative real-time PCR
FBS: Fetal bovine serum
EdU: 5-Ethynyl-2′- deoxyuridine
CCK-8: Cell Counting Kit-8
IHC: Immunohistochemistry
TGF-β: Transforming growth factor-β
